# Transplant with MZ genotype liver: what is the clinical pulmonary picture after 30 years? a case report and review of the literature

**DOI:** 10.1186/s13256-023-04183-7

**Published:** 2023-11-02

**Authors:** Anna Annunziata, Alessandra Di Perna, Ilaria Ferrarotti, Antonietta Coppola, Maria Burricco, Giuseppe Fiorentino

**Affiliations:** 1 Unit of Pathophysiology and Respiratory Rehabilitation, Intensive Care Department, Monaldi Hospital, Naples, Italy; 2 Unit of Pneumology, Latina, Italy; 3 Pneumology Unit, Department of Internal Medicine and Therapeutics, Center for Diagnosis of Inherited Alpha1-Antitrypsin Deficiency, IRCCS San Matteo Hospital Foundation, University of Pavia, 27100 Pavia, Italy

**Keywords:** Alpha 1 antitrypsin, Liver transplant, Lung disease

## Abstract

**Background:**

Alpha-1 antitrypsin, also known as alpha1 proteinase inhibitor, is a protein 90% synthesized by hepatocytes. Alpha-1 antitrypsin deficiency should be suspected if patients have unexplained emphysema or liver disease in the absence of others recognized causes. The diagnosis is based on tests that measure the amount of the enzyme in the blood and confirm by molecular analysis.

**Case presentation:**

We present the case of a man of Caucasian ethnicity, who started experiencing difficulty in breathing 20 years after liver transplantation. After about 30 years since transplantation, an intermediate alpha-1 antitrypsin deficiency is diagnosed with evidence of air trapping, pulmonary emphysema and bronchiectasis.

**Conclusion:**

The presence of a Z-variant synthesized from the donor liver may have contribute to the onset of respiratory disease.

## Introduction

Alpha-1 antitrypsin deficiency is an inherited disease in which a lack of the enzyme alpha-1 antitrypsin can mainly cause lung and liver disease. Adults commonly experience emphysema, with shortness of breath, wheezing and coughing, and some even cirrhosis. Alpha-1 antitrypsin (AAT) is a protein belonging to the serpin superfamily. It is encoded in humans by the SERPINA1 gene, and is also known as an alpha-1 proteinase inhibitor (PI) because it inhibits various proteases. Proteases fragment proteins as part of the normal repair process of lung tissue, if their action is not counterbalanced by AAT, their action can be harmful to lung tissue. More than 90% of circulating AAT is produced by hepatocytes [1].

Alpha-1 antitrypsin deficiency should be suspected if patients have unexplained emphysema, liver disease (in the absence of infection or others recognized causes), panniculitis. The diagnosis is based on tests that measure the amount of the enzyme in the blood and confirm by molecular analysis [1, 2].

## Case description

We describe the case of a 73-year-old male patient, Caucasian ethnicity, never smoker, former office worker, suffering from systemic arterial hypertension and diabetes mellitus. At 43 years of age, he received liver transplant for cirrhosis, secondary to hepatitis B virus infection. At transplant time, patient did not referred pulmonary symptom. No signs of pulmonary disease were found at physical examination. Pulmonary functional tests were normal. CT scan did not reveal abnormalities. ABGs showed normal oxyemia and normal acid–base balance. Alpha 1 antitrypsin serum level was normal (153 mg/dl); it was dosed with a nefelometric methods (Atellica®Siemens).

The orthotopic liver transplant was performed without complications and subsequent clinical follow-up was normal.

Approximately 20 years after liver transplantation, the patient started pneumological check-ups due to the appearance of symptoms characterized by mild dyspnea on exertion and sporadic episodes of bronchitis. For many years, the patient was followed in a general practice clinic and treated with therapy as needed during exacerbations. Due to the progression of symptoms, he received a specialist visit and underwent pulmonary function tests.

**Fig. 1:**
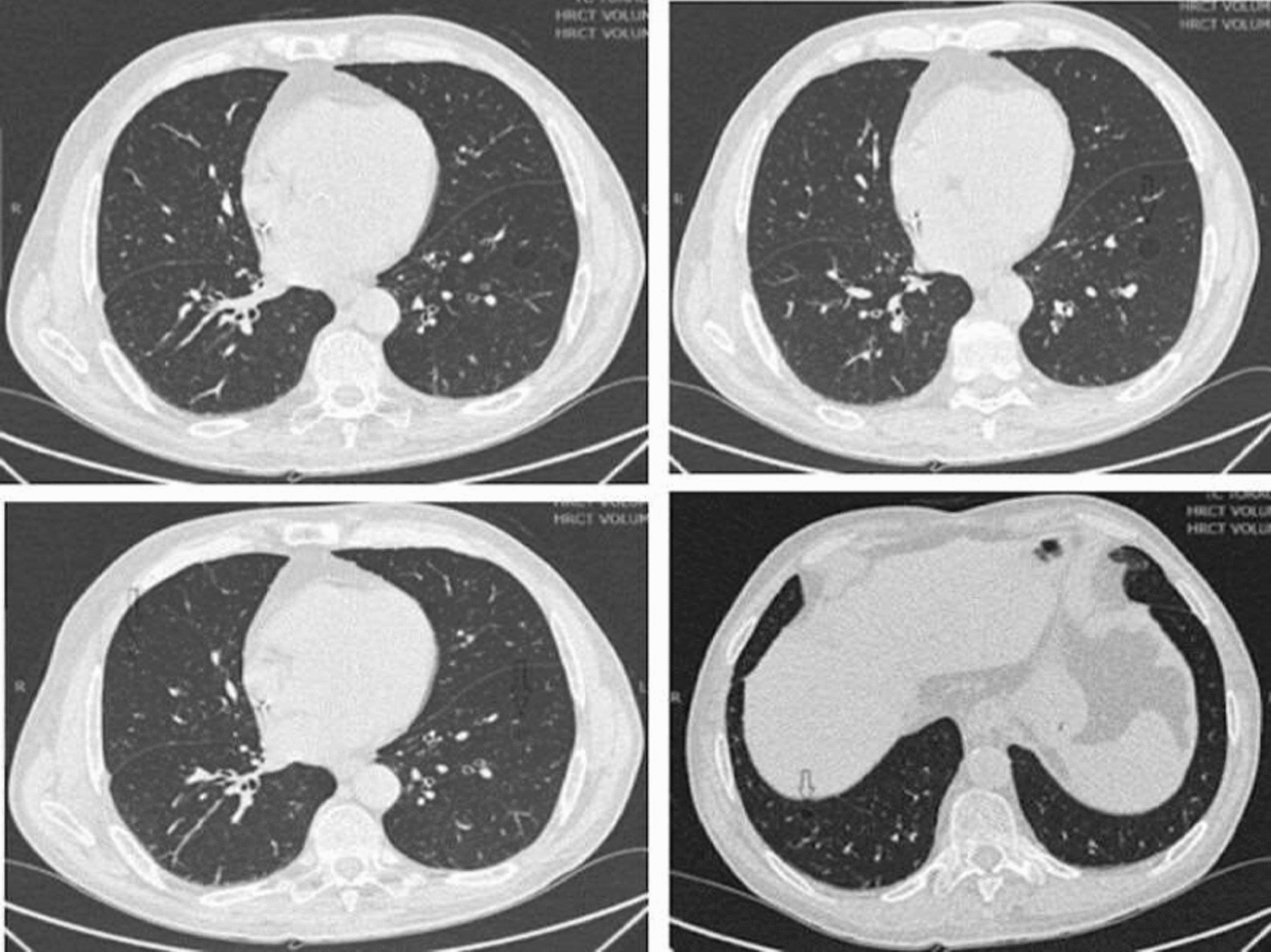
**T**her**e** are diffuse small bronchiectasis, two centrilobular emphysema bubbles in the lateral segment of the lower left lobe, of which the largest with a diameter of about 14 mm and the more peripheral with a diameter of 9.5 mm. A small bubble of emphysema with a maximum diameter of about 7.2 mm is evident in the middle lobe in an almost peripheral location. Another small centrilobular bubble in the posterior segment of the right lower lobe of about 7 mm

Pulmonary function tests revealed mild obstruction, air trapping, with no significant bronchodilator response and reduced diffusing capacity with forced vital capacity (FVC) 4.20 L (119%), forced expiratory volume in 1 s (FEV1) 3.01 L (112%), 8% improvement in broncho dilatory response to drug challenge, FEV1 / FVC ratio 70.6%, total lung capacity (TLC) 11.69 (180%), residual volume (RV) 7,17 L (273%), diffusing capacity (DLCO) 14.12 mL /min/mmHg (58%). The 6 min walking test showed a reduced performance and a modified Medical Research Council (MMRC) dyspnoea of 2. Chest computed tomography (CT) scan showed areas of emphysema in the left lower lobe (Fig. 1).

**Fig. 2:**
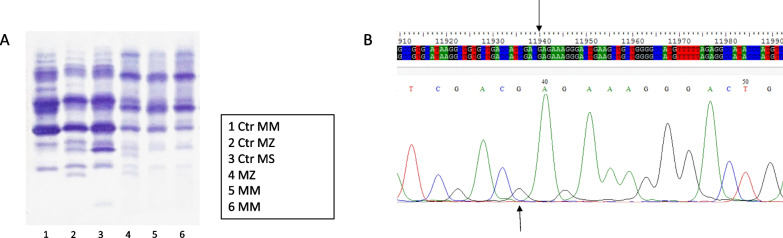
**A** Isoelectrofocusing pattern obtained by Sebia Hydrasys® System on DBS samples. Lines 1–3: standard controls, provided by Hydragel 18 A1AT Isofocusing® kit (Sebia). Lines:4–6 samples. The patient herein described corresponds to samples 4. **B** Sequence (up) and electropherogram(down) of SERPINA1 region of the patient corresponding to position of Z mutation. Arrows show the specific position of nucleotide (G) that should be change into A in the Z variant. The absence of Z mutation is clear both in the sequence of the patient (line 2) when compared to reference sequence (line 1), and in the electropherogram of the patient

The serum AAT level resulted below normal (74 mg/dL with normal values 90–200 mg/dL). Therefore, suspecting an AAT deficiency, a blood sample from the patient was sent to the specialized laboratory in Pavia for further analysis.The phenotype obtained by isoelectrofocusing (IEF) was MZ, showing the presence of both M and Z proteins (Fig. 2A). This data was consistent with the plasma value of AAT previously reported, in accordance to a condition of intermediate AAT deficiency. However, SERPINA1 gene sequencing did not reveal the presence of Z variant, corresponding to a G to A transversion at nucleotide 1096 of cDNA of SERPINA 1 gene (rs28929474) (Fig. 2B) and reported the PI*MM genotype.

## Discussion

Laboratory testing consisting of a combination of quantitative and qualitative (protein phenotyping, genotyping) are used to diagnose the AAT deficiency. Assessment of blood protein concentration and phenotyping are related to protein analysis, whereas molecular analysis such as genotyping and sequencing analyses SERPINA1 gene [3].

A variety of factors can cause discordance among phenotype, genotype, and quantitative AAT results. Some discordant results are related to limitations or errors that occur during the pre-analytic and analytic phases of testing [4].

Discordant results can also be caused by certain clinical characteristics of the patient. A commonly discordant example is caused by AAT replacement therapy, which consists of partially purified AAT-enriched plasma preparations that are administered weekly or biweekly to restore protective level of AAT. The patients with AAT deficiency who are treated with augmentation therapy show a pathological genotype but may have normal serum concentrations and phenotype (corresponding to the infused protein), depending on when the AAT replacement therapy was administered. Also, some clinical conditions may increase or decrease serum AAT concentrations, causing discrepant AAT results. AAT is an acute-phase protein and is released into the circulation following an inflammatory stimulus. AAT sampling during the acute phase in patients with genetic AAT deficiency may result in pathological pheno/genotypes with normal AAT concentration.

In contrast, conditions including liver damage and protein-losing enteropathies can decrease serum AAT concentrations, potentially leading to a normal phenotype/genotype and poor quantitation results in individuals without genetic deficiency of AAT.

Discordant results have also been documented in patients who have received solid-organ transplantations. Patients with AAT deficiency who, for unrelated reasons, have received a bone marrow transplant are reported to have normal genotype, reflecting the bone marrow donor, but a variant phenotype and deficient serum concentrations, reflecting the continued production of variant AAT protein from the patient's liver [5]. Patients with AAT deficiency who have received a liver transplant are reported to show both a normal phenotype and serum concentrations from the healthy donor liver, but they retain a pathological genotype, as described in a case report a few years ago [6]. Unlike subjects with genetic AAT deficiency who have received a liver transplant will display both normal phenotype and serum concentrations from the healthy donor liver but will maintain a pathogenic genotype. Some authors have described changes in AAT phenotype after orthotopic liver transplant, starting 1 day after transplant [7]. Liver transplantation has also been documented to cause discordant phenotype and genotype results of other proteins produced by the liver, including Factor V or cytochrome P450 enzymes [8].

Surprisingly, liver transplant through a donor with undiagnosed AAT deficiency could occur. In such a case, only the complete analysis of AAT, both at the biochemical and genetic level, could provide a reliable and direct diagnosis. In the above case, the patient had a normal genotype (PI*MM) together with a pathological phenotype (MZ). Actually, donor liver produced a deficient amount of protein resulting in an intermediate deficiency of AAT and decrease protection of lungs. Indeed, the presence of impaired respiratory function, areas of emphysema in a patient who had never smoked, in the presence of a reduced serum concentration of AAT led to suspect the presence of AAT deficiency. The onset of clinical deterioration in the patient with intermediate type AAT deficiency has been the subject of debate and controversial opinions for many years. Recently, evidence of lung damage in patient with intermediate deficiency is beginning to emerge [9, 10]. In the light of current knowledge, it is possible to assume that the presence of the Z variant has slowly promoted the onset of lung damage over the years [11].

## Conclusion

In conclusion, AAT deficiency is an under-recognized disorder that can have severe effects on the lungs and liver. Discrepancies in AAT quantitation, phenotype and genotype results may be caused by several clinical factors, blood transfusions, acute-phase response, or bone marrow or liver transplantation. It is therefore important for clinicians to be aware of the various clinical circumstances that can cause discordant AAT results. To the best of our knowledge this is the first case in the literature describing clinical lung disease years after a liver transplant with induction of variant Z synthesis. It is not known, due to the scarcity of data in this area, whether the presence of a Z-variant synthesized from the donor liver may have contributed, as we have hypothesized, to the onset of respiratory disease in a non-smoker.

## Data Availability

Data supporting our findings are available at UOC respiratory pathophysiology, AORN DEI COLLI, Naples, Italy.

## Abbreviations

AAT: Alpha 1 antitripsin
FVC: Forced vital capacity,
FEV 1: Forced expiratory volume in 1s
FEV1: FVC ratio
TLC: Total lung capacity
RV: Residual volume
DLCO: Diffusing capacity
